# Circular RNAs and Hepatocellular Carcinoma: New Epigenetic Players With Diagnostic and Prognostic Roles

**DOI:** 10.3389/fonc.2021.653717

**Published:** 2021-04-20

**Authors:** Kedeerya Aishanjiang, Xin-dong Wei, Yi Fu, Xinjie Lin, Yujie Ma, Jiamei Le, Qiuqin Han, Xuan Wang, Xiaoni Kong, Jinyang Gu, Hailong Wu

**Affiliations:** ^1^ Shanghai University of Medicine and Health Sciences Affiliated Zhoupu Hospital, Department of Collaborative Innovation Center for Biomedicine, Shanghai, China; ^2^ Department of Transplantation, Xinhua Hospital Affiliated to Shanghai Jiao Tong University School of Medicine, Shanghai, China; ^3^ Department of General Surgery, The 81st Hospital Affiliated to Nanjing University of Traditional Chinese Medicine, Nanjing, China; ^4^ Institute of Clinical Immunology, Department of Liver Diseases, Central Laboratory, ShuGuang Hospital Affiliated to Shanghai University of Traditional Chinese Medicine, Shanghai, China; ^5^ Shanghai Key Laboratory of Molecular Imaging, Shanghai University of Medicine and Health Sciences, Shanghai, China; ^6^ Collaborative Innovation Center for Biomedicine, Shanghai University of Medicine & Health Sciences, Shanghai, China

**Keywords:** circular RNAs, hepatocellular carcinoma, biomarkers for diagnosis and prognosis, oncogenic circRNAs, tumor suppressive circRNAs, drug resistance

## Abstract

Hepatocellular carcinoma (HCC) is one of the leading causes of cancer-related death worldwide. Due to the lack of potent diagnosis and prognosis biomarkers and effective therapeutic targets, the overall prognosis of survival is poor in HCC patients. Circular RNAs (circRNAs) are a class of novel endogenous non-coding RNAs with covalently closed loop structures and implicated in diverse physiological processes and pathological diseases. Recent studies have demonstrated the involvement of circRNAs in HCC diagnosis, prognosis, development, and drug resistance, suggesting that circRNAs may be a class of novel targets for improving HCC diagnosis, prognosis, and treatments. In fact, some artificial circRNAs have been engineered and showed their therapeutic potential in treating HCV infection and gastric cancer. In this review, we introduce the potential of circRNAs as biomarkers for HCC diagnosis and prognosis, as therapeutic targets for HCC treatments and discuss the challenges in circRNA research and chances of circRNA application.

## Introduction

Hepatocellular carcinoma (HCC) is ranked as the sixth most common neoplasm and the third leading cause of cancer-related death worldwide (1). Curative treatments, including liver transplantation, liver resection, and ablation, are only available for early stage HCCs. But due to the absence of specific symptoms at early stages and the lack of early diagnostic biomarkers, most HCC patients are diagnosed at an advanced stage and not eligible for curative treatments (2). So far, the tyrosine kinase inhibitors (sorafenib and lenvatinib) and the combination of atezolizumab (an anti–PD-L1 antibody) with bevacizumab (a vascular endothelial growth factor inhibitor) are the only approved first-line systemic treatment for advanced HCCs (3–5). Moreover, although 5-year overall survival (OS) rate reaches up to 50%, recurrence occurs in more than 70% HCC patients after curative surgery (6), which severely impairs the prognosis of HCCs. Therefore, development of early diagnostic and/or prognostic biomarkers and identification of novel therapeutic targets are urgently required for improving HCC outcomes.

**Figure 1:**
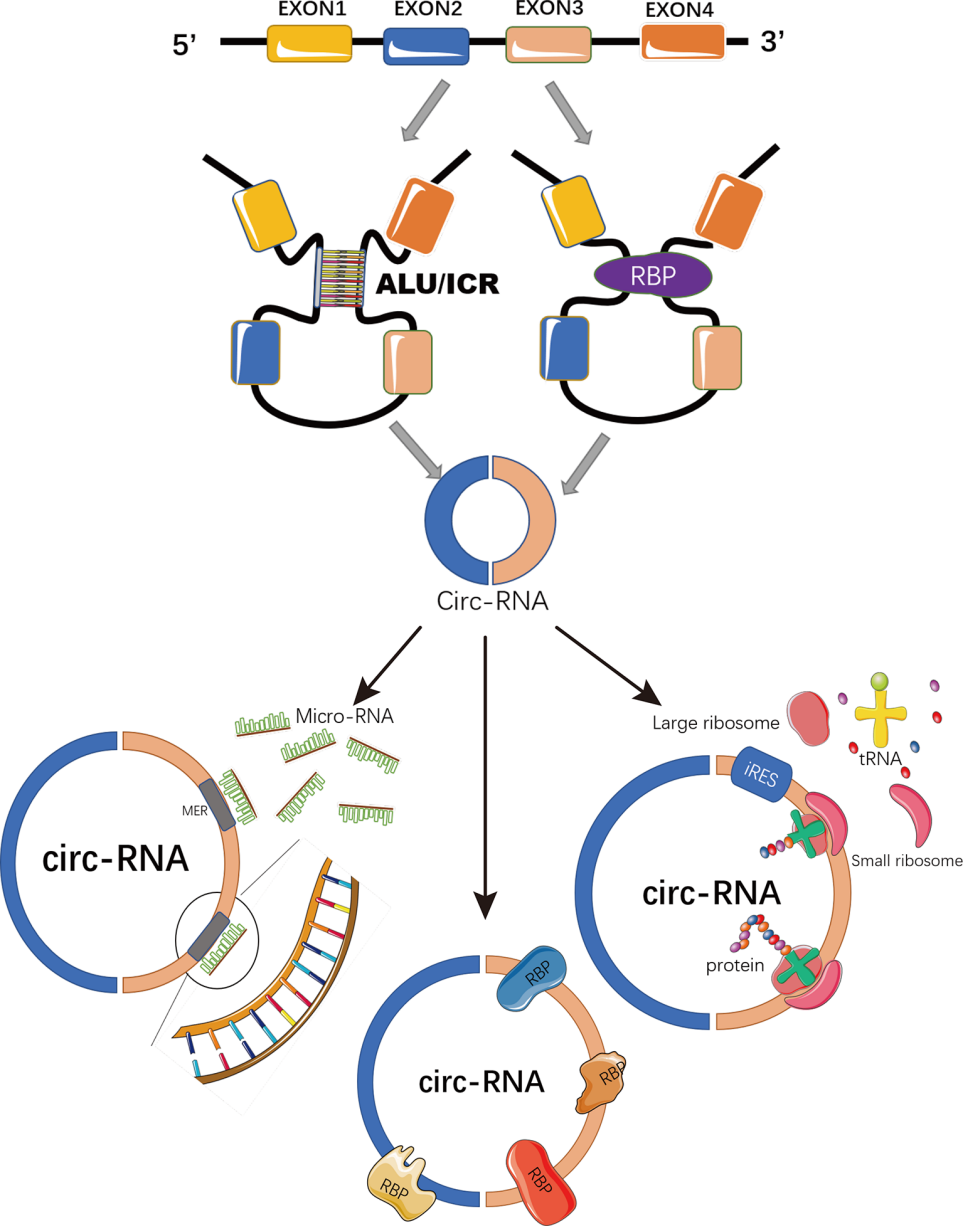
Schematic representation of circRNA biogenesis and functions. CircRNAs are covalently closed loop structures generated by back-splicing of pre-mature transcripts mediated by either Alu repeats, inverted complementary repeats (ICR), or RNA binding proteins (RBP). CircRNAs could function as miRNA sponges to block miRNA-mediated inhibition on target genes; or as scaffolds, recruiters or decoys of some RNA binding proteins to regulate the function of the associated proteins. Some circRNAs containing IRES and ORFs serve as RNA template for translation.

Circular RNAs (circRNAs) are a novel class of non-coding RNAs generated from back-splicing of pre-mature transcripts by forming covalently closed loop structures without 5′-caps or 3′-polyadenylated tails (7, 8) (**Figure 1**). Although most circRNAs were originally recognized as by-products of aberrant splicing (9), accumulating evidence has suggested their involvement in various physiological processes and pathological conditions, such as viral infection, sepsis, cardiovascular disease, diabetes, and aging and regenerative medicine (9–13). By functioning as miRNA sponges (14), acting as protein decoys, scaffolds and recruiters (15) or serving as RNA templates for short peptide synthesis (16), circRNAs play important roles in regulating gene expression and signaling transduction (8) (**Figure 1**).

Recent emerging evidence has demonstrated that circRNAs are closely associated with tumor initiation and progression (17–20). Firstly, deregulation of circRNAs has been confirmed in many types of cancers, including breast cancer (21, 22), lung cancer (23, 24), prostate cancer (25), colorectal cancer (26), gastrointestinal cancers (27), ovarian cancer (28), thyroid cancer (29), gynecologic cancers (30), and hepatocellular carcinoma (31); Secondly, some circRNAs have been demonstrated to play either oncogenic (32, 33) or tumor suppressive (34, 35) roles in affecting multiple cancer hallmarks, including deregulating cellular energetic, self-sufficiency in growth signals, insensitivity anti-growth signals, evading cell death, limitless replicative potential, substained angiogenesis, tissue invasion and metastasis (36, 37). Moreover, given the facts that circRNAs are more resistant to exoribonuclease degradation due to their lack of free 5′- and 3′-ends (38) and are abundant in body fluids, such as saliva, blood, and urine (39–41), they have been increasingly recognized as promising tumor biomarkers (42). This review introduces recently circRNAs identified with biomarker and therapeutic potentials for HCC diagnosis, prognosis and treatments, describes circRNAs related to drug resistance and discusses the challenges in circRNA research and chances of circRNA application.

## CircRNAs as Non-Invasive Diagnosis Biomarkers of HCCs

Early diagnosis is critical for the improvement of HCC prognosis and outcomes. The serum alpha-fetoprotein (AFP) (43), alpha-fetoprotein-L3 (AFP-L3) (44) and Des-gamma-carboxy prothrombin (DCP) (45) are the most commonly used non-invasive circulating biomarkers for HCC diagnosis in clinical practice, but limited evidence supports their contribution to HCC early detection or improvement in HCC outcomes (46). Therefore, identifying novel biomarkers for HCC early diagnosis is urgently required. CircRNAs are dysregulated in HCCs (47), and more resistant to nuclease degradation compared to linear RNAs due to their covalently closed loops (38), which make them potential biomarkers for HCC diagnosis. Here, we mainly introduce some recent studies indicating that circRNAs are promising non-invasive circulating diagnosis biomarkers of HCCs.

Zhang et al. demonstrated that circRNA_104075 was positively regulated by HNF4a, a HCC-promoting transcription factor, and highly expressed in HCC tissues and serum samples. CircRNA_104075 showed greater predictive performance (AUC: 0.973, sensitivity: 0.960; specificity: 0.983) for HCCs than AFP (AUC: 0.750, sensitivity: 0.693; specificity: 0.683), AFP-L3 (AUC: 0.766, sensitivity: 0.772; specificity: 0.633) and DCP (AUC: 0.771, sensitivity: 0.703; specificity: 0.750) (48).

By comparing the serum circRNA profiles between 36 healthy controls, 60 chronic hepatitis C (CHC) and 68 HCC patients, Matboli et al. found up-regulation of hsa_circ_000224 and down-regulation of hsa_circ_001565 and hsa_circ_000520 in the serum samples of HCC patients compared to those in healthy controls and CHC patients (49). The predictive performance for HCCs of hsa_circ_001565 (AUC: 0.839, sensitivity: 0.703; specificity: 0.750), hsa_circ_000520 (AUC: 0.943, sensitivity: 0.971; specificity: 0.896) and hsa_circ_000224 (AUC: 0.974, sensitivity: 0.956; specificity: 0.927) were better than that of serum AFP (AUC: 0.726, sensitivity: 0.779; specificity: 0.823) (49). Combing the three circRNAs showed remarkably high sensitivity (1.000) but did not improve the specificity (0.833). The authors failed to show the AUC value for the combined three circRNAs in diagnosing HCCs (49).

By analyzing differentially expressed circRNAs (DECs) in plasma between 40 healthy controls and 71 HCC patients, Sun et al. recently identified a three-circRNA signature, including hsa_circ_0004001, hsa_circ_0004123, and hsa_circ_0075792, which are highly expressed in HCC patients (50). This 3-circRNA signature showed a potent diagnosis value (AUC: 0.89, sensitivity: 0.905; specificity: 0.781) (50). In this study, the authors did not compared the diagnosis value of the 3-circRNA signature with that of AFP, but the AUC, sensitivity and specificity values of the 3-circRNA signature may slightly better than those of AFP from other studies (48, 49).

Comparison of circulating circRNAs, in 5 HCC patients before and after hepatectomy, 5 HBV-positive chronic hepatitis (CH) patients and 5 healthy controls, identified that the serum levels of hsa_circ_0009582, hsa_circ_0037120, and hsa_circ_0140117 were high in HCCs and decreased after hepatectomy (51). Moreover, a combination of those three circRNAs with serum AFP greatly improved the diagnosis potential for HCC in a training set (20 HCCs vs. 20 CH patients; AUC: 0.988, sensitivity: 0.960; specificity: 1.015) and a validation set (180 HCCs vs. 180 CH patients; AUC: 0.955, sensitivity: 0.915; specificity: 0.994) (51).

Down-regulation of hsa_circ_0001445 (circSMARCA5) in HCCs was identified in 3 cohorts of 208 pairs of HCC and corresponding adjacent noncancerous liver (ANL) tissues, and circSMARCA5 overexpression inhibited HCC growth and metastasis *in vitro* and *in vivo (* 52). By comparing plasma circSMARCA5 levels in 103 healthy controls, 117 hepatitis (hepatitis B and C), 143 cirrhosis and 135 HCC patients, Li et al. found that plasma circSMARCA5 decreases gradually but significantly from healthy controls, hepatitis, cirrhosis to HCC patients (53). It worth noting that, in patients whose AFP levels were less than 200 ng/ml, circSMARCA5 showed satisfying accuracy in distinguishing HCCs from hepatitis (AUC = 0.847, sensitivity: 0.721; specificity: 0.882) and cirrhosis (AUC = 0.706, sensitivity: 0.721; specificity: 0.660) patients, suggesting circSMARCA5 as a putative biomarker for HCCs with low AFP levels (53).

Recently, a large-scale, multicenter study identified a plasma circRNA panel (CircPanel) containing three circRNAs (hsa_circ_0000976, hsa_circ_0007750, and hsa_circ_0139897), which had greater accuracy than AFP in distinguishing individuals with HCC from Non-HCC (54). The performance of CircPanel vs. AFP was AUC 0.863 *vs.* 0.790 (*p*=0.036) in the training set (53 healthy controls, 52 with CHB, 50 with liver cirrhosis and 158 with HCC), AUC 0.843 *vs.* 0.747 (*p*=0.011) in Validation Set 1 (152 HCC patients, 50 healthy controls, 54 CHB patients and 50 HBV-induced liver cirrhosis patients), and AUC 0.864 *vs.* 0.769 (*p<0.001*) in Validation Set 2 (290 HCC patients, 76 healthy controls, 80 CHB patients and 80 HBV-induced liver cirrhosis patients) (54). Moreover, CircPanel showed a higher accuracy than AFP in diagnosis of small-HCC (solitary, diameter ≤ 3 cm) (AUC of CircPanel *vs.* AFP: 0.862 *vs.* 0.680, *p=0.001* in the training set; 0.838 *vs.* 0.699, *p=0.011* in Validation Set 1 and 0.851 *vs.* 0.738, *p=0.009* in Validation Set 2) (54). More significantly, CircPanel has been identified to be able to diagnose AFP-negative HCC and AFP-negative Small-HCC (54).

## CircRNAs As Prognosis Biomarkers of HCCs

Recurrence occurs in more than 70% HCC patients post curative surgery (6). Even for the most effective liver transplantation (LT), the recurrence rate is near 20% (55). Although the TNM staging system of the American Joint Committee on Cancer (AJCC), the Barcelona Clinic Liver Cancer (BCLC) classification, and the Cancer of the Liver Italian Program (CLIP) staging system, have been employed to evaluate the prognosis of HCC patients (56), their prognostic predictive performance was unsatisfactory partly because those assessments do not take the critical and complicated molecular pathogenesis of HCCs into account (57). Recent studies demonstrated that some circRNAs could serve as prognostic biomarkers for HCCs.

Hsa_circ_0000267 was up-regulated in HCC tumor tissues and positively associated aggressive clinicopathological features such as tumor size and TNM stage (58). Multivariable analysis identified enhanced hsa_circ_0000267 as an independent prognostic factor for the OS in HCC patients (58).

Chromosomal amplification at 7q21–7q31 was closely related to tumor recurrence in various types of cancers including HCCs (59–62). By assessing the expression of 43 putative circRNAs in this chromosomal region in 4 pairs of matched HCC and normal tissues, Huang et al. identified that hsa_circ_0082002 (circMET) was the most significantly and consistently up-regulated circRNA in HCCs (63). By evaluating circMET expression in 90 paired HCC and normal tissues and in a tissue microarray (TMA) consisting 209 HCC tissues, levels of circMET were significantly and positively correlated with microvascular invasion, absent tumor encapsulation, multiple tumors and late HCC stage. Multivariate analysis identified circMET as an independent predictor for both OS and postoperative recurrence in HCC patients (63).

Sun et al. have demonstrated that hsa_circ_0027345 (circLRIG3), a nucleus-enriched circRNA, was highly expressed in HCCs and positively associated with aggressive clinicopathological features, such as tumor size, vascular invasion, Edmondson’s grade and later TNM stage, and was identified as an independent risk prognostic factor for OS in HCC patients (64).

The down-regulation of hsa_circ_0001727 (circZKSCAN1) in HCCs were determined by two independent groups (65, 66). Expression of circZKSCAN1 was associated with multiple clinicopathologic factors, such as tumor numbers, cirrhosis, vascular invasion, microscopic vascular invasion, or tumor grade (65), and was determined as an independent and significant factor affecting both OS and RFS in HCCs (66).

In addition to serving as a non-invasive biomarker, decreased expression of circSMARCA5 in HCC tumor tissues was significantly correlated with poorer tumor differentiation, more advanced tumor stage, larger tumor size and presence of microvascular invasion. Multivariate analysis demonstrated that circSMARCA5 expression level was an independent risk factor for OS and RFS in HCCs (52).

A recent study has demonstrated that an up-regulated plasma circRNA, hsa_cic_0005397, was positively correlated with tumor size (p = 0.020) and TNM stage (p = 0.006) (67). Meanwhile, a dynamic monitoring of plasma hsa_cic_0005397 in HCC patients who had undergone surgical resection revealed that plasma hsa_cic_0005397 level was drastically dropped in HCC patients after operation, but prominently elevated in recurrent or metastatic HCC patients, suggesting that plasma hsa_cic_0005397 could serve as a prognostic biomarker for post-operational recurrence and metastasis in HCC patients (67). Moreover, the plasma hsa_cic_0005397 level was negatively associated with OS in HCC patients (67).

However, due to the limited sample size and the lack of multicenter validation, the diagnosis and prognosis potential of those circRNAs should be further determined in the future.

## Oncogenic and Tumor Suppressive CircRNAs in HCCs

Like many other non-coding RNAs, such as lncRNAs and miRNAs, circRNAs have been demonstrated to function as either oncogenes or tumor suppressors in HCCs.

By comparing circRNA expression profiles in primary HCCs with or without post-surgery pulmonary metastasis, Hu et al. identified that hsa_circ_0085616 (circASAP1) is highly expressed in HCCs with greater metastatic potential (31). Mechanistically, circASAP1 functions as a sponge for miR-326 and miR-532-5p and enhances the expression of CSF-1 and MAPK1, resulting in an increase in intra-tumor infiltration of tumor associated macrophages and activation of the MAPK-ERK signaling pathway (31). Consistent with this study, Li et al. also demonstrated that circASAP1 plays an oncogenic role in HCCs possibly by activating the β-catenin, ERK and AKT signaling pathways (68).

Through bioinformatics analysis and experimental validation, Li et al. demonstrated that up-regulation of hsa_circ_0074854 (circMAT2B) in HCCs was correlated with poor prognosis and could serve as an independent prognostic factor (69). By functioning as a sponge of miR-338-3p, circMAT2B released miR-338-3p-mediated inhibition on PKM2, resulting in enhanced glycolysis, proliferation and metastasis in HCCs (69).

In addition to acting as a miRNA sponge, some circRNAs have been reported to function as protein decoys, scaffolds and recruiters to promote HCC progression. Wang et al. demonstrated that hsa_circ_102034 (circRHOT1) was highly expressed in HCCs and promoted proliferation, migration and invasion in HCC cells by acting as a protein recruiter to recruit TIP60 onto the promoter region of *NR2F6* to initiate *NR2F6* expression (70).

By performing a circRNA microarray assay specifically targeting human circRNA splicing sites in seven paired HCC tumor and normal samples, Han et al. demonstrated that hsa_circ_0007874 (circMTO1) was significantly decreased in HCCs and correlated with poor prognosis of HCC patients (71). CircMTO1 could serve as a miR-9 sponge to release miR-9–mediated inhibition on p21, resulting in inhibition on HCC growth both *in vitro* and *in vivo (* 71).

Compared to oncogenic circRNAs, the tumor suppressive circRNAs are less studied probably due to their low expression in HCCs. Only countable circRNAs have been identified as tumor suppressors in HCCs. For example, circSMARCA5, a down-regulated circRNA in HCC, has been identified as a tumor suppressor for HCC progression. Over-expression of circSMARCA5 retained miR-17-3p and miR-181b-5p to release their common target, TIMP3, a well-known tumor suppressor in HCCs, resulting in enhanced HCC growth and metastasis both *in vitro* and *in vivo (* 52, 72). By functioning as a decoy of fragile X mental retardation protein (*FMBP*), an RNA binding protein, circZKSCAN1 prevented the binding of FMBP with cell cycle and apoptosis regulator 1 (*CCAR1*) mRNA and in turn inhibited *CCAR1* expression and CCAR1-mediated activation of the Wnt/β-catenin signaling pathway, resulting in compromised stemness in HCC cells (66).

## CircRNAs Related to Drug RESISTANCE in HCCs

Advanced HCCs are not legible for curative treatments. Although chemotherapy, targeted therapy and immunotherapy are commonly employed for those advanced HCCs, the therapeutic efficacy is unsatisfied due to low objective response rate and acquired resistance (73–75). In addition to playing oncogenic or tumor suppressive roles in HCC progression, some circRNAs have been recently reported to be related to drug resistance in HCC treatments.

For HCC chemotherapy, hsa_circ_0001001 (circFBXO11), an up-regulated circRNA in HCCs, could serve as a sponge for miR-605 to induce FOXO3-mediated ABCB1 expression, resulting in HCC oxaliplatin resistance (76). CircRNA_101505 was down-regulated in cisplatin-resistant HCC tissues and cell lines, and over-expression of circRNA_101505 trapped miR-103 and relieved its target, oxidored-nitro domain-containing protein 1 (NOR1), a putative tumor suppressor in HCCs (77), consequently resulting in cisplatin sensitization (78).

For sorafenib-mediated targeted therapy, Wu et al. have identified thousands of deregulated circRNAs in sorafenib-resistant HCC cells compared to sorafenib-sensitive cells (79). Yang et al. have demonstrated that hsa_circ_0058124 (circFN1) was up-regulated in both sorafenib-resistant HCC tumor tissues and cell lines (80). Overexpression of circFN1 conferred HCC cell sorafenib resistance by restricting miR-1205–mediated E2F1 inhibition (80).

For PD1 antibody mediated immunotherapy, circMET has been demonstrated to confer HCC cells resistance to anti-PD1 treatment by enhancing the immunosuppressive tumor microenvironment (63). Mechanistically, circMET functions as a sponge of miR-30-5p to induce SNAIL-mediated Dipeptidyl 4 (DPP4) expression, resulting in degradation of CXCL10, an important chemokine promoting intra-tumor infiltration of effector T cells, and leading to subsequent resistance to anti-PD1 treatment (63).

## Exosomal circRNAs in HCC Progression

Exosome is a type of nano-sized secreted vesicles, which are present in all body fluids under both physiological and pathological conditions (81). By carrying various biomolecules, including proteins, nucleic acids, lipids, and transferring from one cell to another, exomes play an important role in mediating intercellular communication (81). In 2015, Li, et al. for the first time demonstrated the presence of abundant circRNAs in exosomes (82). By RNA-seq analysis, they demonstrated enrichment of circRNAs in exosome compared to producer cells (82). A number of exosomal circRNAs have been identified to function as either diagnosis/prognosis biomarkers or oncogenic/tumor suppressive factors in HCCs (83, 84). For example, an exosomal circRNA, hsa_circ_0070396, was identified as a better diagnostic biomarker than AFP in distinguishing HCCs from healthy controls (AUC: 0.8574 vs. 0.781), and differentiating early HCC patients (with BCLC are 0 or A) from advanced ones (with BCLC are B+C) (AUC: 0.7132 vs. 0.6535) (85). Higher circulating exosomal circAKT3 was identified in HCC patients compared to healthy controls, and positively correlated with tumor recurrence rates and mortality, suggesting that circAKT3 could serve as a prognostic biomarker of HCCs (86). A recent study has demonstrated that three exosomal circPTGR1 isoforms secreted from HCC cells with higher metastasis potential to promote the metastasis of HCC cells with lower metastasis potential *via* regulating the miR449a-MET pathway (87). Su et al. have demonstrated that circRNA Cdr1as competitively bound to miR-1270 to upregulate AFP level, thereafter accelerating proliferative and migratory abilities of HCC cells (88). Meanwhile, exosome-transmitted circRNA Cdr1as stimulated malignant behaviors of surrounding normal cells and finally contributed to the progression of HCC (88). An interesting study showed that a pro-invasive exosomal circRNA-100338 secreted from HCC cells could transfer to HUVECs to affect cell proliferation, angiogenesis, permeability, and vasculogenic mimicry (VM) formation ability of HUVECs, and in turn promote HCC metastasis (89).

Instead of transporting exosomal circRNAs from HCC cells to surrounding normal cells, the exosomal circRNA can also be generated and transported from surrounding normal tissues to HCC cells, resulting in altered HCC progression. For example, an exosomal circRNA, circ-0051443, which was generated in normal cells and transferred to HCC cells, could inhibit HCC progression by competitive binding to miR-331-3p, resulting in enhanced apoptosis and cell cycle arrest in HCC cells (90).

It is clear that exosomal circular RNAs play a significant role in HCC progression. Therefore, identifying novel exosomal circRNAs and understanding their biological functions can not only help us better understand the new mechanisms of potential HCC development, but also improve the clinical diagnosis, prognosis and treatment of HCCs.

We have summarized all the circRNAs mentioned in this review in **Table 1**.

**Table 1:** Summary of mentioned circRNAs with diagnosis, prognosis and therapeutic potential in HCCs.

Classification	CircRNA Symbols in Paper	Current circBase_ID	Host genes	Potential Application	Reference
Non-invasive diagnosis biomarkers	circRNA_104075	_	_	A serum biomarker for HCC diagnosis	(48)
	hsa_circ_001565 hsa_circ_000520 hsa_circ_000224	hsa_circ_0000064 hsa_circ_0000221 hsa_circ_0000737	*B4GALT2* *VIM* *C17orf107*	Serum biomarkers for HCC diagnosis	(49)
	_	hsa_circ_0004001, hsa_circ_0004123, hsa_circ_0075792	*CLK1* *ETV6* *KDM1B*	As a serum-derived three-circRNA signature for HCC diagnosis	(50)
	_	hsa_circ_0009582 hsa_circ_0037120 hsa_circ_0140117	*RERE* *RHBDF1* *CNKSR2*	As circulating biomarkers predicting the occurrence of HBV-related HCCs	(51)
	circSMARCA5	hsa_circ_0001445	*SMARCA5*	As a circulating biomarker to accurately distinguish HBV-related HCCs from hepatitis and cirrhosis patients with low AFP levels	(52, 53)
	CircPanel	hsa_circ_0000976 hsa_circ_0007750 hsa_circ_0139897	*HPCAL1* *RABGGTA* *MTM1*	Be able to diagnose AFP-negative HCC and AFP-negative Small-HCC	(54)
Prognosis Biomarkers	circSMARCA5	hsa_circ_0001445	*SMARCA5*	As an independent prognostic factor for the OS and RFS in HCC patients	(52)
	hsa_circ_0000267	hsa_circ_0000267	*FAM53B*	As an independent prognostic factor for the OS in HCC patients	(58)
	circMET	hsa_circ_0082002	*MET*	As an independent prognostic factor for the OS and RFS in HCC patients	(63)
	circLRIG3	hsa_circ_0027345	*LRIG3*	As an independent prognostic factor for the OS in HCC patients	(64)
	circZKSCAN1	hsa_circ_0001727	*ZKSCAN1*	As an independent prognostic factor for the OS and RFS in HCC patients	(65, 66)
	hsa_cic_0005397	hsa_cic_0005397	*RHOT1*	A prognostic biomarker for post-operational recurrence and metastasis in HCC patients	(67)
Oncogenic circRNAs	circASAP1	hsa_circ_0085616	*ASAP1*	Therapeutic target for HCC metastasis and immunotherapy	(31, 68)
	circMAT2B	hsa_circ_0074854	*MAT2B*	Therapeutic target for HCC progression	(69)
	circRHOT1	hsa_circ_102034	*RHOT1*	Therapeutic target for HCC progression	(70)
Tumor suppressive circRNAs	circMTO1	hsa_circ_0007874	*MTO1*	Therapeutic target for HCC progression	(71)
	circSMARCA5	hsa_circ_0001445	*SMARCA5*	Therapeutic target for HCC progression	(52, 72)
	circZKSCAN1	hsa_circ_0001727	*ZKSCAN1*	Therapeutic target for HCC progression	(66)
CircRNAs related to drug resistant	circMET	hsa_circ_0082002	*MET*	Therapeutic target for HCC anti-PD1 resistance	(63)
	circFBXO11	hsa_circ_0001001	*FBXO11*	Therapeutic target for HCC oxaliplatin resistance	(76)
	CircRNA_101505	hsa_circ_0002891	*PDIA3*	Therapeutic target for HCC cisplatin sensitization	(77)
	circFN1	hsa_circ_0058124	*FN1*	Therapeutic target for HCC sorafenib resistance	(80)
Exosomal CircRNAs	hsa_circ_0070396	hsa_circ_0070396,	*NUDT9*	A better diagnostic biomarker than AFP in distinguishing HCCs	(85)
	circAKT3	hsa_circ_0000199	*AKT3*	A prognostic biomarker of HCCs	(86)
	circPTGR1	hsa_circ_0008043 hsa_circ_0003731 hsa_circ_0088030	*PTGR1*	Promote the metastasis of HCC cells with lower metastasis potential	(87)
	CircRNA_Cdr1as	hsa_circ_0001946	*Cdr1as*	Accelerate proliferative and migratory abilities of HCC cells	(88)
	circRNA-100338	hsa_circ_0000130	*SNX27*	Promote HCC metastasis through regulating angiogenesis	(89)
	circ-0051443	hsa_circ_0051443	*TRAPPC6A*	Inhibit HCC progression by enhancing apoptosis and cell cycle arrest in HCC cells	(90)

## Challenges and Chances of CircRNAs

With the advent of high-throughput sequencing and high-efficiency big data analysis, circular RNAs have been emerged as a novel class of non-coding RNAs in eukaryotes. Accumulating evidence indicates that circRNAs are novel non-coding players the involved in various biological processes and diseases. However, some fundamental questions regarding the function and regulation of circRNAs remain unknown.

First, the biological role of circRNAs is less defined. Only less than 3% (> 7000 circRNAs) of recorded circRNAs (295,526 circRNAs integrated from circBase, circNet, and circRNAdb) (91) were curated functional (92), which at least in part suggests that the most circRNAs may be the non-functional by-products of RNA splicing events. In a previous study, Guo et al. claimed that compared to mRNAs, most circRNAs are less abundant, less cell-type specific and less conserved (93). Moreover, ribosome profiling provides no evidence for their translation (93).

Second, serving as miRNA sponges may not be the general role of circRNAs. Although abundant studies support the role of circRNAs as miRNA sponges in regulating gene expression and executing their biological functions, the existence of numerous circRNAs in *P. falciparum* and *S. cerevisiae (* 94, 95), who lack known siRNA and miRNA pathways, suggests that as miRNA sponges may not be a general role for most circRNAs. This is consistent with a previous study showing that there is no particular enrichment of AGO2 binding on exons involved in circRNAs compared to their neighboring linear exons (93).

Third, the *cis* and *trans* regulators for circRNAs remain illuminated. Some studies demonstrated that the significantly longer introns and the inverted repeat sequence bracketing the regions that produce circRNAs are essential *cis* regulators for circRNA formation in humans and flies (38, 96–98). Meanwhile, canonical splicing signals are required for the circularization of most circRNAs (93, 99), suggesting that canonical spliceosomal machinery may serve as *trans* regulators for circRNA formation. However, accumulating strong evidence indicates that the expression of circRNAs does not simply correlate with their linear host genes (100, 101), suggesting that circRNA formation is a complicated process which may be regulated by additional circRNA-specific *cis* and *trans* regulators.

Although it remains controversial on the general roles of circRNAs, most functional circRNAs identified so far have been demonstrated to serve as miRNA sponges in regulating gene expression. By employing this feature, some artificial circRNAs have been engineered to sequester miRNAs relevant in human disease and shown promising potential for application in molecular medicine and biology. For example, an artificial circRNA carrying an array of miRNA-122 binding sites could sequester liver specific miR-122 from HCV RNA, resulting in inhibition on HCV viral protein production and HCV replication (102). Liu et al. generated an artificial circRNA sponge for miR-21, an oncomiR in many types of cancers, to block miR-21–mediated proliferation in gastric carcinoma cells (103). Therefore, in companion of growing understanding of circRNAs, expended application of circRNAs could be established for both research and clinic purposes.

## Conclusion and Prospect

In summary, circRNAs are emerging as a class of novel non-coding RNAs participating diverse physiological processes and pathological conditions. Although it remains controversial on the general roles of circRNAs, accumulating evidence indicates that circRNAs are functional by serving as either miRNA sponges, protein decoys, or translational templates. Like other types of non-coding RNAs, deregulation of circRNAs has been demonstrated in HCCs. A number of deregulated circRNAs have been identified as either oncogenic or tumor suppressive regulators in HCC progression. Moreover, some deregulated circRNAs could serve as non-invasive circulating biomarkers for HCC early diagnosis with great specificity and sensitivity superior to clinically used serum AFP. Meanwhile, some circRNAs have been involved in drug resistance in HCC treatments. In this review, we summarized the recent findings of circRNAs in HCC diagnosis, prognosis, progression and drug resistance and proposed that circRNAs may have great potential to serve as novel biomarkers for HCC early diagnosis, prognosis and therapeutic targets for HCC treatments.

Despite the advancements achieved in the circRNA field, several fundamental questions remain elusive. Given the fact that only a few percentage functional circRNAs (< 3%) were identified from the great circRNA popularity, whether circRNAs are a group of functional non-coding RNAs is still an open question. The existence of numerous circRNAs in species deficient with RNAi and miRNA pathways raises the question whether as miRNA sponges is the general role of circRNAs or just is an exception. Although some studies have demonstrated that the formation of circRNAs depends on the *cis* elements, such as the inverted repeats, and the *trans* regulators, such as spliceosomes, of linear host genes, the lack of correlation at expression levels and cell-type specificity between circRNAs and their linear host genes strongly suggests the existence of circRNA specific regulators. Therefore, novel techniques and concepts regarding those fundamental questions are warranted in the future to expand our understanding of circRNAs and improve our investigation on circRNAs.

## Author Contributions

KA, X-DW, YF, XL, YM, JL, XW, and QH performed extensive literature search and discussion. KA and X-DW drafted the manuscript. XK, JG, and HW edited the manuscript. All authors contributed to the article and approved the submitted version.

## Funding

This work was supported by grants from the National Natural Science Foundation of China (31870905 to HW, 82072645 and 81772507 to JG, 82070633 and 81873582 to XK), the Scientific Program of Shanghai Municipal Health Commission (SHWJ2019211 to HW), the Shanghai Municipal Education Commission-Gaofeng Clinical Medicine Grant Support (No. 20191910), the Foundation for Shanghai Jiao Tong University for SMC-morning Star Youth Scholars Program, the Clinical Research Plan of SHDC (SHDC2020CR3005A), the Program of Medical Engineering Cross Research Fund of Shanghai Jiao Tong University (YG2017MS50), Clinical innovation project of Xinhua Hospital Affiliated to Medical College of Shanghai Jiaotong University (19XHCR02A), “Teaching incentive plan” project of Xinhua Hospital Affiliated to Medical College of Shanghai Jiaotong University (XH001.006.010.020), National Health and Family Planning Commission medical and health science and technology development research center key projects (NHC2018RWS01007), the Construction project of Shanghai Key Laboratory of Molecular Imaging(18DZ2260400), Shanghai Municipal Education Commission (Class II Plateau Disciplinary Construction Program of Medical Technology of SUMHS, 2018-2020), and the Key Program of National Natural Science Foundation of China (grant 81830052).

## Conflict of Interest

The authors declare that the research was conducted in the absence of any commercial or financial relationships that could be construed as a potential conflict of interest.

## Abbreviations

AFP, alpha-fetoprotein; AFP-L3, alpha-fetoprotein-L3; AJCC, the American Joint Committee on Cancer; ANL, adjacent noncancerous liver; AUC, Area Under Curve; BCLC, the Barcelona Clinic Liver Cancer; CCAR1, cell cycle and apoptosis regulator 1; CH, chronic hepatitis; CHC, chronic hepatitis C; circRNAs, Circular RNAs; CLIP, the Cancer of the Liver Italian Program; CSF-1, colony-stimulating factor 1; CXCL10, C-X-C motif chemokine ligand 10; DCP, Des-gamma-carboxy prothrombin; DECs, differentially expressed Circular RNAs; DPP4, SNAIL-mediated Dipeptidyl 4; FMBP, fragile X mental retardation protein; HCC, Hepatocellular carcinoma; HCV, Hepatitis C Virus; HNF4a, hepatocyte nuclear factor 4 alpha; LT, liver transplantation; MAPK1, mitogen-activated protein kinase 1; NOR1, Oxidored-nitro domain-containing protein 1; OS, Overall Survival; RFS, relapse-free survival; TMA, tissue microarray; Cdr1as, Cerebellar degeneration-related protein 1 antisense RNA; HUVECs, human umbilical vein endothelial cells; AKT3, AKT serine/threonine kinase 3.
